# Targeted chemotherapy overcomes drug resistance in melanoma

**DOI:** 10.1101/gad.333864.119

**Published:** 2020-05-01

**Authors:** Jingyin Yue, Roberto Vendramin, Fan Liu, Omar Lopez, Monica G. Valencia, Helena Gomes Dos Santos, Gabriel Gaidosh, Felipe Beckedorff, Ezra Blumenthal, Lucia Speroni, Stephen D. Nimer, Jean-Christophe Marine, Ramin Shiekhattar

**Affiliations:** 1 Department of Human Genetics, Sylvester Comprehensive Cancer Center, University of Miami Miller School of Medicine, Miami, Florida 33136, USA;; 2 Laboratory for Molecular Cancer Biology, Oncology Department, KULeuven, 3000 Leuven, Belgium;; 3 Center for Cancer Biology, VIB, 3000 Leuven, Belgium;; 4 Department of Biochemistry, University of Miami, Miami, Florida 33136, USA;; 5 Department of Medicine, Sylvester Comprehensive Cancer Center, University of Miami Miller School of Medicine, Miami, Florida 33136, USA

**Keywords:** DNA damage response, drug resistance, cellular dormancy, MAP kinase signaling, protein phosphatase 2A, small molecule inhibitor

## Abstract

The emergence of drug resistance is a major obstacle for the success of targeted therapy in melanoma. Additionally, conventional chemotherapy has not been effective as drug-resistant cells escape lethal DNA damage effects by inducing growth arrest commonly referred to as cellular dormancy. We present a therapeutic strategy termed “targeted chemotherapy” by depleting protein phosphatase 2A (PP2A) or its inhibition using a small molecule inhibitor (1,10-phenanthroline-5,6-dione [phendione]) in drug-resistant melanoma. Targeted chemotherapy induces the DNA damage response without causing DNA breaks or allowing cellular dormancy. Phendione treatment reduces tumor growth of BRAF^V600E^-driven melanoma patient-derived xenografts (PDX) and diminishes growth of NRAS^Q61R^-driven melanoma, a cancer with no effective therapy. Remarkably, phendione treatment inhibits the acquisition of resistance to BRAF inhibition in BRAF^V600E^ PDX highlighting its effectiveness in combating the advent of drug resistance.

## Abstract (precis)

In this study, Yue et al. describe a therapeutic strategy termed “targeted chemotherapy” that involves depleting PP2A or inhibiting it using a small molecule inhibitor, phendione, in drug-resistant melanoma. The authors show phendione induces DNA damage response without causing DNA breaks or inducing cellular dormancy, therefore blocking tumor growth of BRAF mutant and NRAS mutant melanomas.

The mitogen-activated protein kinase (MAPK) pathway is the most frequently mutated signaling cascade in cancer (Davies et al. 2002). The MAPK pathway is initiated by binding of growth factors such as epidermal growth factor (EGF) to their receptor tyrosine kinase (Peyssonnaux and Eychène 2001). The signal is propagated through the RAS GTPase and the RAF-MEK and ERK kinases, which leads to a potent induction of immediate early genes (IEGs) (Murphy et al. 2002). A large number of cancers including lung, colon, skin, pancreas, and multiple forms of leukemia harbor oncogenic mutations that promotes constitutively active MAPK signaling. These include mutations in FLT3, EGFR, KRAS, HRAS, NRAS, and BRAF (Davies et al. 2002; Malumbres and Barbacid 2003; Muzumdar et al. 2017; Patnaik 2017). The constitutive activation of the MAPK pathway promotes the proliferation and survival of cells and paves the path for cancer initiation and progression (McCubrey et al. 2007).

Tremendous strides have been made in the development of compounds that specifically target mutant kinases, and in particular, components of the MAPK pathway (Sawyers 2004; Gharwan and Groninger 2016). Targeting RAS has proven to be extremely challenging and consequently very few therapeutic options exist for RAS mutant cancer, which includes ∼30% of melanomas (Devitt et al. 2011; Jakob et al. 2012). Inhibitors of oncogenic BRAF, which occurs in ∼50% of melanoma patients (Cancer Genome Atlas 2015), have led to unprecedented clinical responses in patients with BRAF mutant metastatic melanoma. However, the clinical benefit of such agents is limited by genetic and/or nongenetic adaptive mechanisms that lead to intrinsic or acquired resistance (Manzano et al. 2016). Additionally, cancer cells can escape the effects of conventional chemotherapeutic agents through differentiation, de-differentiation into a slow cycling cell population, and/or entering a senescence state, which are collectively referred to as cellular dormancy (Yeh and Ramaswamy 2015). These clinical observations highlight the need for improved molecular strategies, combinations and/or sequential therapies that target cancer cells without allowing growth arrest. Here we show that protein phosphatase 2A (PP2A) inhibition provides a unique opportunity for cancer therapy by inducing the DNA damage response (DDR) without causing DNA breaks or promoting cellular dormancy.

## Results

### Targeting DNA damage response phosphatases in melanoma

**Figure 1.**
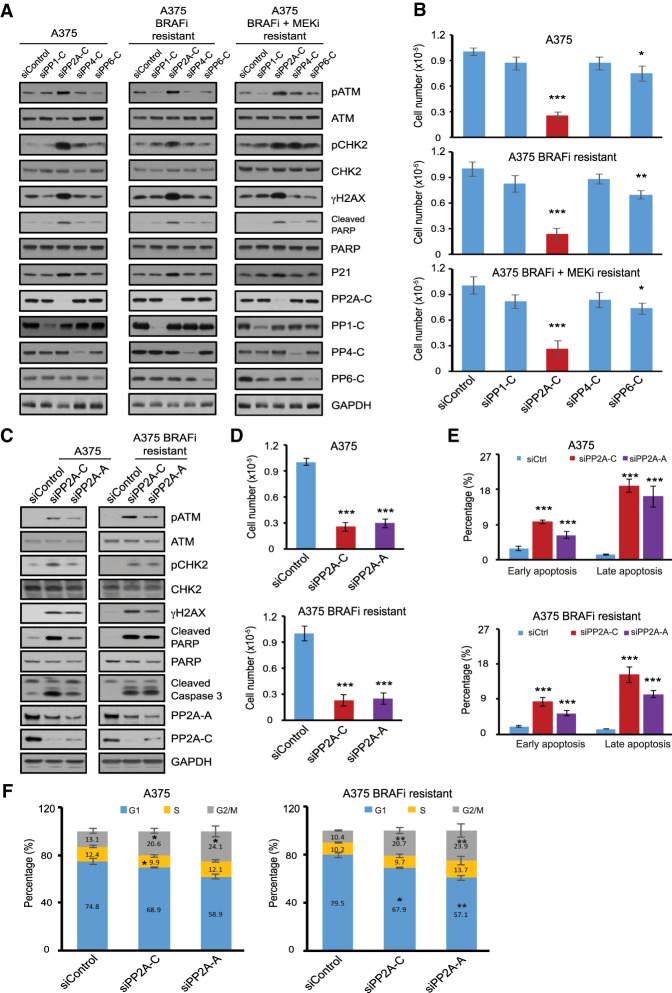
Targeting DDR phosphatases in melanoma activates DNA damage signaling and inhibits cell proliferation. (*A*) Immunoblots of cell lysates collected 3 d after siRNA transfection from parental A375, A375 BRAFi-resistant, and A375 BRAFi + MEKi-resistant cells. The cells were transfected with control siRNA or siRNA targeting the catalytic subunits of protein phosphatases PP1-C (siRNA pool against α, β, and γ isoforms), PP2A-C (siRNA pool against α and β isoforms), PP4-C or PP6-C. (*B*) Proliferation assay of parental A375, A375 BRAFi-resistant, and A375 BRAFi + MEKi-resistant cells. The number of viable cells was counted 3 d after siRNA transfection as shown in *A*. Data are shown as average ± SD of three independent experiments. (*C*) Immunoblots of cell lysates collected 3 d after siRNA transfection from parental A375 and A375 BRAFi-resistant cells. The cells were transfected with control siRNA or siRNA targeting the catalytic subunits of protein phosphatases PP2A-C (siRNA pool against α and β isoforms) or scaffolding regulatory subunit PP2A-A (siRNA pool against α and β isoforms). (*D*) Proliferation assay of parental A375 and A375 BRAFi-resistant cells. The number of viable cells was counted 3 d after siRNA transfection as shown in *C*. Data are shown as average ± SD of three independent experiments. (*E*) Quantification of cell apoptosis measured by Annexin V staining flow cytometry. Parental A375 and A375 BRAFi-resistant cells were transfected with siRNAs as described in *C* and apoptotic cells were measured 3 d after transfection. Results were presented as average ± SEM (*n* = 2). (*F*) Cell cycle analysis for A375 and A375 BRAFi-resistant cells performed 3 d after transfection with siRNAs as described in *C*. Graphs show the percentage of cells in each phase of the cell cycle. Data shown are average ± SD of three independent experiments. (*) *P* < 0.05; (**) *P* < 0.01; (***) *P* < 0.001, by unpaired two-tailed Student's *t*-test.

While conventional chemotherapy is effective for a variety of cancers, its utilization has been hampered by the development of a growth arrest commonly known as tumor dormancy (Gao et al. 2017). Paradoxically, melanomas that acquire resistance to BRAF inhibition display increased sensitivity to conventional chemotherapeutic DNA-damaging agents upon drug discontinuation (Kong et al. 2017). We sought to devise a strategy that exploits the DNA damage vulnerability of melanoma cells resistant to MAPK therapy while evading cell cycle arrest. We reasoned that targeting the protein phosphatases that regulate DNA damage signaling (Freeman and Monteiro 2010) may provide such an opportunity. We assessed the role of key phosphatases that regulate the DDR by the activation of ATM and its downstream targets, CHK2 and γH2AX, the main components of DNA damage signaling. We also monitored poly(ADP-ribose) polymerase (PARP) cleavage, a measure of cellular apoptosis (Ivana Scovassi and Diederich 2004). We depleted catalytic subunits of a number of protein phosphatases, including PP1-C (the catalytic subunit of protein phosphatase 1 [PP1]), PP2A-C (the catalytic subunit of PP2A), PP4-C (the catalytic subunit of protein phosphatase 4 [PP4]), and PP6-C (the catalytic subunit of protein phosphatase 6 [PP6]) and examined the induction of DNA damage signaling in three cell lines, including the parental A375 melanoma cell line harboring BRAF^V600E^ mutation, the previously described A375-derived cell line (Yue et al. 2017) with resistance to BRAF inhibitor (A375 BRAFi-resistant) and another A375-derived cell line with resistance to both BRAF inhibitor and MEK inhibitor (trametinib [MEKi]; A375 BRAFi + MEKi-resistant) (Supplemental Fig. S1A–D). Intriguingly, depletion of PP2A catalytic subunits (PP2A-C, both α and β isoforms) led to a specific and potent activation of ATM and induction of the DDR pathway (pCHK2 and γH2AX) as well as PARP cleavage in all three cell lines (Fig. 1A). PP2A-C depletion also displayed the greatest decrease in cellular proliferation across the three cell lines tested, consistent with activation of DNA damage signaling (Fig. 1B). Importantly, these observations were recapitulated by knockdown of the PP2A regulatory subunit (PP2A-A [the scaffolding regulatory subunit of PP2A] both α and β isoforms) (Fig. 1C,D). Finally, reduction of PP2A catalytic and regulatory subunits increased the proportion of cells in apoptosis and in G2/M phase of the cell cycle (Fig. 1E,F; Supplemental Fig. S1E,F). These results attest to the critical role of PP2A in the activation of the DNA damage response, inhibition of cell proliferation, and cell cycle arrest in both parental and drug-resistant A375 melanoma cell lines.

### Phendione is a small molecule inhibitor of PP2A

**Figure 2.**
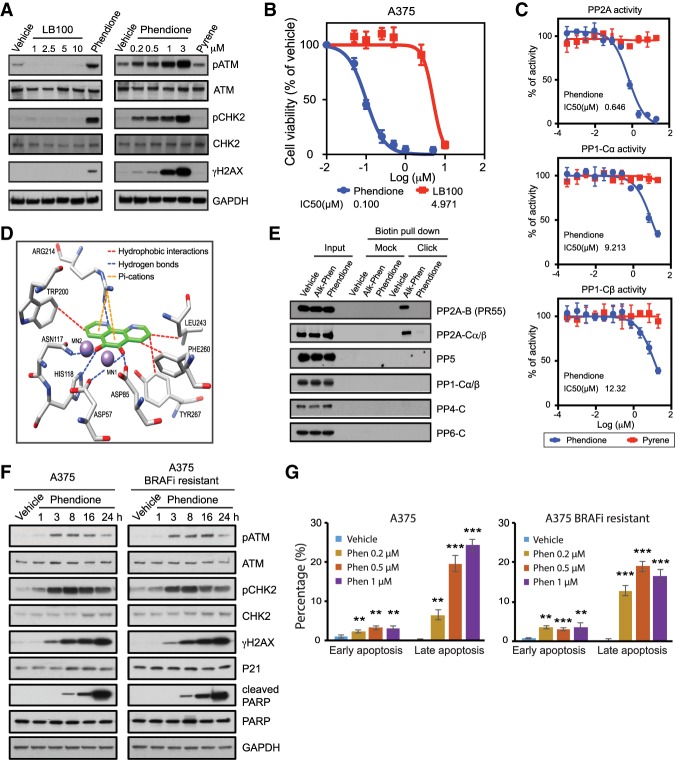
Phendione is a small molecule inhibitor targeting PP2A. (*A*) Immunoblots of A375 cell lysates collected after 3 h of drug treatment as indicated; i.e., increasing concentrations of LB100 compared with 0.2 µM phendione (*left*) and increasing concentrations of phendione compared with 3 µM pyrene (*right*). DMSO was used as negative control (vehicle). (*B*) Growth inhibition curves of A375 cells and IC50 values after treatment with phendione or LB100 for 96 h. Data are presented as mean ± SD of three independent experiments. (*C*) Phosphatase activity assay for PP2A core enzyme (complex of regulatory subunit Aα and catalytic subunit Cα; *top*), PP1 catalytic subunit Cα (*middle*) and PP1 catalytic subunit Cβ (*bottom*) incubated with phendione or pyrene at indicated concentrations. Data shown are average ± SD of two independent experiments. IC50s (micromolar) were calculated and is shown as an *inset* in each graph. (*D*) Binding model of phendione to PP2A. Docking pose of phendione (green) interacting with the catalytic residues of PP2A-Cα (PPP2CA) (white) within 5 Å around the ligand. Oxygens (red), nitrogens (blue), and manganese ions (purple) are shown. Dotted lines represent intermolecular polar contacts. Hydrogens are omitted for clarity. (*E*) Immunoblots for phendione derivative pull down. (*F*) Immunoblots of A375 and A375 BRAFi-resistant cell lysates after treatment with 0.2 µM phendione or vehicle for indicated time. (*G*) Quantification of cell apoptosis measured by Annexin V staining flow cytometry. Parental A375 and A375 BRAFi-resistant cells were treated with vehicle or increasing concentration of phendione as indicated in the panels for 24 h before apoptotic cells were measured by flow cytometry. Results were presented as average ± SD (*n* = 3). (**) *P* < 0.01; (***) *P* < 0.001, by unpaired two-tailed Student's *t*-test.

We next aimed to find a small molecule inhibitor of PP2A to induce the DNA damage response and inhibit melanoma cellular proliferation. LB100 was previously reported as a small molecule inhibitor of PP2A (Lu et al. 2009). Treatment of A375 cells with LB100 failed to induce the DDR at concentrations up to 10 µM, and the IC50 of LB100 for inhibition of A375 proliferation was 5 µM (Fig. 2A,B). In search of a small molecule inhibitor of PP2A that mimics PP2A depletion and subsequent DDR, we examined phendione (1,10-phenanthroline-5,6-dione), a phenanthroline derivative reported to inhibit phosphatase activity (Urbanek et al. 2001). Notably, a number of studies identified that phenanthroline and its derivatives promote cytotoxicity in cancer cells (Deegan et al. 2006; Pivetta et al. 2014). Indeed, phendione specifically inhibited recombinant PP2A (a core two-subunit enzyme of PP2A-Cα and PP2A-Aα), but not PP1-Cα or PP1-Cβ, in an in vitro phosphatase assay. Pyrene, a flat aromatic molecule, was used as a control (Fig. 2C). Molecular modeling supported the association of phendione with key residues of PP2A-Cα catalytic cleft with an estimated free energy of binding of −6.24 kcal/mol (Fig. 2D; Supplemental Fig. S2A). To confirm the direct association of phendione with PP2A, we developed alkyne-phendione to perform copper-catalyzed Click chemistry for biotin conjugation (Supplemental Fig. S2B). These experiments revealed that alkyne–phendione specifically interacts with PP2A following the Click reaction and biotin pull-down, confirming a direct association of phendione with PP2A (Fig. 2E). Importantly, in contrast to LB100, nanomolar concentrations of phendione were sufficient to induce the DDR and diminish the proliferation of A375 cells (Fig. 2A,B). Additionally, a time course and dose-escalation analysis of phendione revealed that BRAFi-resistant A375 cells displayed a similar induction of DDR, PARP cleavage, and apoptotic response to that of the parental line (Fig. 2F,G; Supplemental Figs. S1D, S2C–F). While we found differences in efficacy between the enzyme- and cell-based assays, such differences have been observed previously and are generally attributed to limitations of the in vitro assay's reliance on recombinant enzymes that may be partially folded or need additional regulatory subunits to display the full extent of their enzymatic activity (Krzyzosiak et al. 2018). Taken together, these results pinpoint phendione as an inhibitor of PP2A whose effects manifest in increased ATM phosphorylation and activation of the DNA damage response, leading to impaired growth of melanoma cells with acquired resistance to MAPK inhibitors.

### Phendione inhibits the proliferation of melanoma with intrinsic resistance to MAPK inhibitors

**Figure 3.**
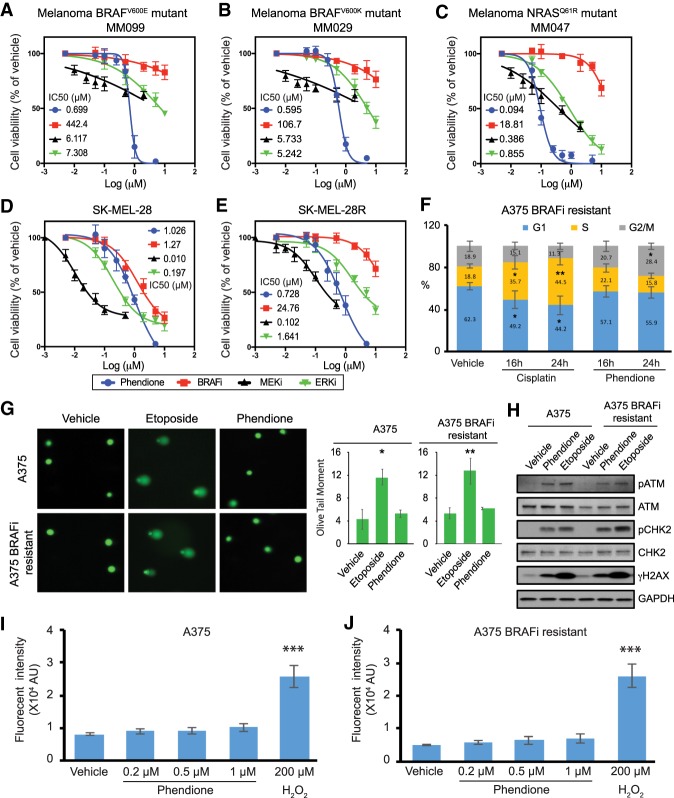
Phendione inhibits the proliferation of melanoma with intrinsic resistance to MAPK inhibitors. (*A–E*) Panels show growth inhibition curves and IC50 values. Primary patient-derived melanoma cell lines with BRAF^V600E^, BRAF^V600K^, and NRAS^Q61R^ mutations (*A–C*), and human melanoma cell line SK-MEL28 (*D*) and its BRAF inhibitor (dabrafenib)-resistant line SK-MEL28R (*E*) were treated with phendione or inhibitors of the MAPK pathway for 96 h. Data are presented as mean ± SD of three independent experiments. (*F*) Cell cycle analysis for A375 BRAFi-resistant cells. Cells were treated with 2.5 µM cisplatin, 0.2 µM phendione, or vehicle for the indicated time. Graph shows the percentage of cells in each phase of the cell cycle. Data shown are average ± SD of three independent experiments. (*G*) Comet assay to measure DNA damage. Representative images are shown at the *left* and quantification results are shown at the *right*. Data are presented as mean ± SD of three independent experiments. (*H*) Immunoblots of whole-cell lysate collected from comet assay. (*I*,*J*) Quantification of reactive oxygen species (ROS) production measured by flow cytometry in A375 (*I*) and A375 BRAFi-resistant (*J*) cells. Hydroxyl peroxide (H_2_O_2_; 200 µM) was used as positive control. Data are presented as means ± SEMs of three independent experiments. (*) *P* < 0.05; (**) *P* < 0.01; (***) *P* < 0.001. Statistical analysis was performed using unpaired two-tailed Student's *t*-test.

We next assessed phendione growth inhibitory activity in a variety of primary patient-derived cell lines exhibiting a mesenchymal-like/invasive phenotype, which confers intrinsic resistance to inhibitors of the MAPK pathway (Kemper et al. 2014; Verfaillie et al. 2015; Titz et al. 2016; Shaffer et al. 2017). MM099 BRAF^V600E^ mutant melanoma cells were resistant to MAPK inhibition as expected, though they showed a strong decrease in cell viability following phendione treatment (Fig. 3A). Similarly, other invasive cell lines such as MM029 (BRAF^V600K^) and MM047 (NRAS^Q61R^) were exquisitely sensitive to phendione while their responsiveness to MAPK inhibitors was muted (Fig. 3B,C). BRAF^V600E^ SK-MEL-28R cells were resistant to BRAFi, MEKi, and ERKi yet showed a robust response to phendione that was comparable with the parental SK-MEL-28 cell line (Fig. 3D,E). Therefore, phendione can overcome both intrinsic and acquired drug resistance in melanoma resulting in potent inhibition of tumor cell proliferation.

### Phendione mechanism of action is distinct from that of conventional chemotherapeutic agents

To assess mechanistic differences between phendione and other DNA break-inducing chemotherapeutics, we measured cell cycle progression of BRAFi-resistant cells treated with phendione or cisplatin. In contrast to cisplatin, which triggered a block in S phase progression, phendione treatment resulted in a small, yet significant, increase in the fraction of cells in the G2/M phase of the cell cycle (Fig. 3F; Supplemental Fig. S3A). The parental A375 and BRAFi/MEKi-resistant cell lines exhibited a similar response to phendione and also induced a small increase in the G2/M population (Supplemental Fig. S3A,B; data not shown). Comet assays confirmed that unlike etoposide, another DNA damage inducing agent, phendione did not cause DNA breaks, rather it activated DNA damage signaling (Fig. 3G,H). Importantly, a flow cytometry assay revealed that phendione treatment did not promote the generation of reactive oxygen species (Fig. 3I,J; Supplemental Fig. S3C). Finally, the cytotoxicity of phendione was assessed in a panel of melanoma cell lines and normal cells, including primary human melanocytes (HEMn). While nontransformed lines were relatively resistant to phendione, most melanoma cells exhibited low micromolar IC50 (Supplemental Fig. S3D). Additionally, primary human melanocytes required a higher dose of phendione to induce DNA damage signaling and apoptosis (Supplemental Fig. S3E). These results indicate that phendione activates ATM and the consequent DNA damage response pathway by a mechanism of action that is distinct from cisplatin and etoposide, both of which produce physical DNA breaks.

### Phendione does not chelate intracellular cations

**Figure 4.**
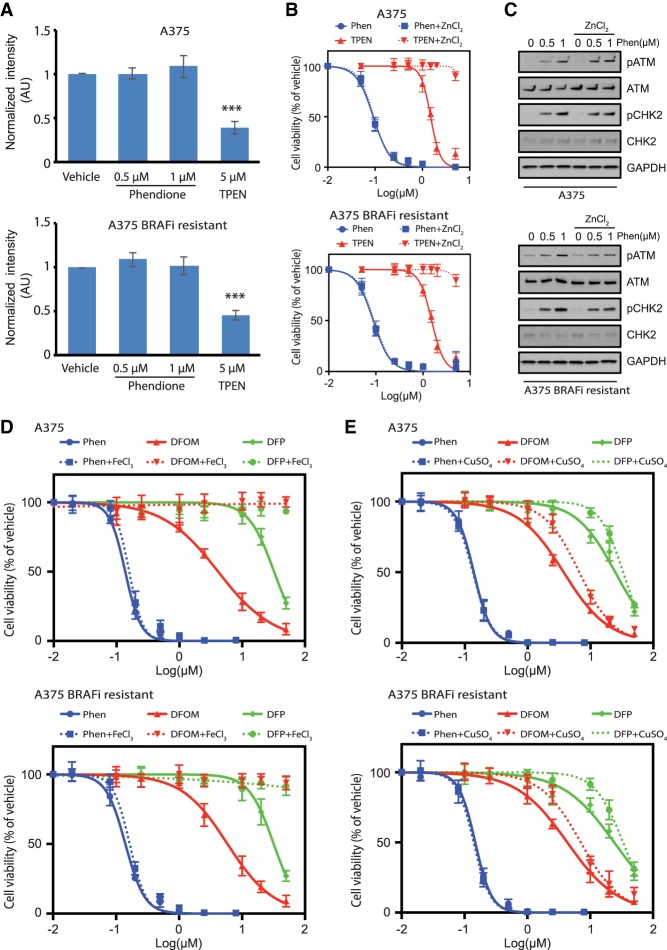
Phendione does not chelate intracellular cations. (*A*) Quantification of fluorescent intensity in the cells labeled with zinc indicator by flow cytometry. A375 (*top*) and A375 BRAFi-resistant (*bottom*) cells were treated with vehicle, phendione or cell-permeable zinc chelator TPEN for 3 h. At the end of treatment, 20 µM zinquin ethyl ester was added into medium to stain Zn^2+^ in live cells. Unstained cells were used as negative control for flow cytometry. (***) *P* < 0.001 by unpaired two-tailed Student's *t*-test. (*B*) Growth inhibition curves of A375 (*top*) and A375 BRAFi-resistant (*bottom*) cells treated with phendione or zinc chelator TPEN prepared in regular medium or medium containing 15 µM ZnCl_2_. (*C*) Immunoblot of whole-cell lysate collected after the treatment of vehicle or phendione prepared in regular medium or medium containing 15 µM ZnCl_2_ as indicated. (*D*,*E*) Growth inhibition curves of A375 (*top*) and A375 BRAFi-resistant (*bottom*) cells after 96 h of treatment with phendione or ion chelators DFOM or DFP prepared in regular medium or medium containing 50 µM FeCl_3_ (*D*) or 10 µM CuSO_4_ (*E*).

To assess whether the effects of phendione are mediated through zinc chelation, we measured intracellular zinc in parental and BRAFi-resistant cells using a flow cytomery assay. Unlike TPEN [N,N,N′,N′-tetrakis (2-pyridinylmethyl)-1,2-ethanediamine], a known membrane-permeable zinc chelator, phendione did not reduce intracellular zinc levels (Fig. 4A). Accordingly, in contrast to TPEN treatment, where the addition of ZnCl_2_ rescued cell viability, the DDR and cytotoxicity incurred by phendione treatment was not rescued with ZnCl_2_ (Fig. 4B,C). To evaluate whether impaired cellular viability was mediated through chelation of other metals, FeCl_3_ and CuSO_4_ were each administered alongside phendione. While the addition of these salts mitigated the cytotoxicity of DFOM (deferoxamine mesylate) and DFP (deferiprone) chelators, the loss of viability and induction of DNA damage signaling persisted in parental and BRAFi-resistant cells treated with phendione (Fig. 4D,E; Supplemental Fig. S4A,B). Moreover, cell viability was not restored by the addition of MgCl_2_, CaCl_2_, or NaCl to cells treated with phendione (Supplemental Fig. S4C,D). Taken together, these findings indicate that unlike conventional ion chelating agents, neither the activation of the DNA damage response nor the inhibition of cellular proliferation by phendione is mediated through the chelation of intracellular cations.

### PP2A inhibition activates MAPK response in BRAF resistant melanoma

**Figure 5.**
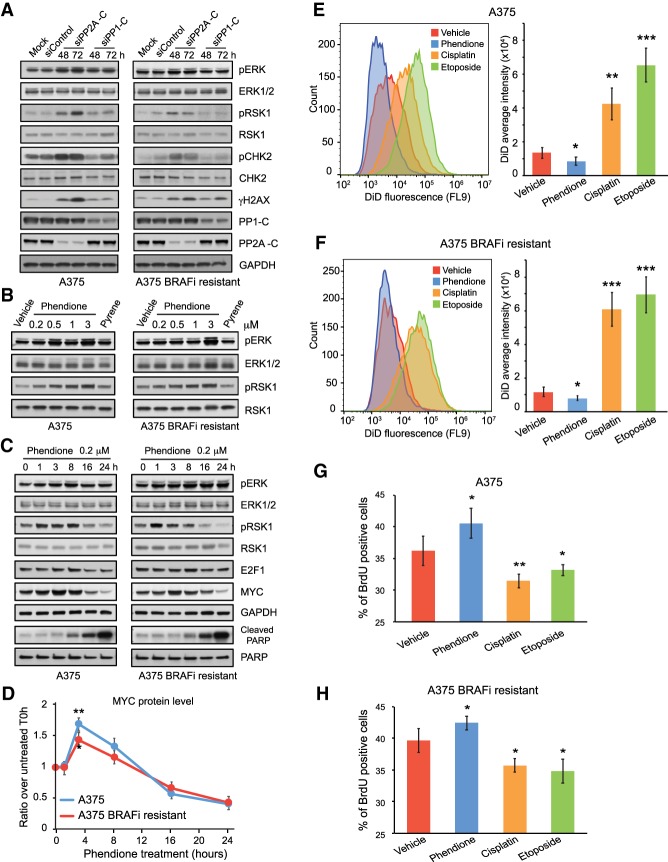
PP2A inhibition transiently promotes cell proliferation leading to evasion from dormancy. (*A–C*) Immunoblots of cell lysate collected from A375 and A375 BRAFi-resistant cells after 2 and 3 d of siRNA transfection (*A*), 3 h of drug treatment (*B*), and time-course treatment with 0.2 µM phendione (*C*) as indicated in each panel, respectively. siRNAs used for transfection include control siRNA, siRNA targeting the catalytic subunits of protein phosphatases PP1-C (siRNA pool against α, β, and γ isoforms) and PP2A-C (siRNA pool against α and β isoforms). (*D*) Quantification of MYC protein level from immunoblots after time-course treatment with 0.2 µM phendione in A375 and A375 BRAFi-resistant cells. GAPDH protein level was used as internal control to perform normalization. Data shown are mean ± SEM of three independent experiments. (*E*,*F*) Histogram and quantification of fluorescent signal from Vybrant DiD cell-labeling dye detected by flow cytometry. (*G*,*H*) Quantification of incorporated bromodeoxyuridine (BrdU) measured by flow cytometry. Data shown are mean ± SD of two independent experiments. (*) *P* < 0.05; (**) *P* < 0.01; (***) *P* < 0.001. Unpaired two-tailed Student's *t*-test was used for statistical analysis.

PP2A has been suggested to regulate MAPK signaling (Junttila et al. 2008). Therefore, we explored the possibility that phendione induces cell death by promoting cell cycle progression amidst elevated DNA damage signaling via hyperactivation of the MAPK cascade. Indeed, depletion of PP2A led to an enhancement of ERK1/2 activity in both the parental and BRAFi-resistant melanoma cells (Fig. 5A). Consistent with the effects observed after PP2A depletion, inhibition of PP2A using phendione similarly led to activation of ERK1/2 in a dose-dependent manner (Fig. 5B). Critically, a temporal analysis using 200 nM phendione treatment showed up-regulation of the cell cycle transcriptional activators MYC and E2F1 within the initial time course (1–8 h) followed by their degradation and induction of cell death at 24 h, as shown by PARP cleavage (Fig. 5C,D). Thus, in melanoma cells resistant to BRAF inhibition, phendione treatment or PP2A depletion activates the MAPK cascade and cell cycle entry concurrently with DNA damage signaling and apoptosis.

To assess cellular dormancy, we asked whether 3 h of acute treatment with phendione or conventional chemotherapeutic agents affects the proliferative potential of cancer cells 14 d later. We measured proliferation of cells after treatment with phendione, cisplatin, etoposide, or vehicle using incorporation of the Vybrant DiD cytoplasmic membrane marker or BrdU (bromodeoxyuridine) labeling of DNA (Fig. 5E–H). Remarkably, while treatment with conventional chemotherapeutic agents induced a potent S-phase arrest, phendione treatment increased cell proliferation, suggesting a distinct molecular mechanism that prevents cell cycle arrest (Fig. 5E–H). Indeed, the BrdU incorporation rate demonstrates that acute treatment with phendione increased the proliferative capacity of melanoma cells (Fig. 5G,H). These results indicate that phendione, which activates the MAPK response and DNA damage signaling, maintains cell proliferation and enhances the window in which drug-resistant cells could succumb to DNA damage.

### Phendione-induced growth inhibition is ATM-dependent

**Figure 6.**
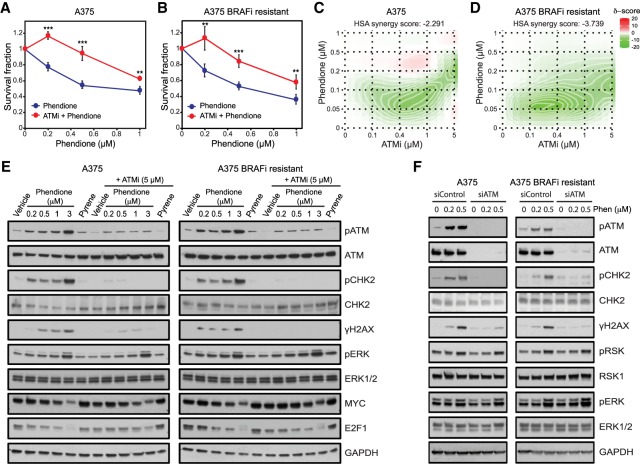
Phendione-induced growth inhibition is ATM dependent. (*A*,*B*) Colony formation assay of A375 (*A*) and A375 BRAFi-resistant (*B*) cells treated with phendione or phendione in combination with ATMi (5 µM) for 3 h. Data are shown as mean ± SD of three independent experiments. (**) *P* < 0.01; (***) *P* < 0.001, by two-way ANOVA with Sidak's multiple comparisons test. (*C*,*D*) Synergy maps for A375 (*C*) and A375 BRAFi-resistant (*D*) cells showing the effect of combined treatment with phendione and ATMi at indicated concentrations for 96 h. The 2D synergy matrix was generated with SynergyFinder software using HAS model. (*E*) Immunoblots of A375 and A375 BRAFi-resistant cell lysates after treatment with phendione, pyrene (3 µM), or vehicle for 3 h in the presence or absence of ATM inhibitor (5 µM). (*F*) Immunoblots of A375 and A375 BRAFi-resistant cell lysates after 3 d of siRNA transfection as indicated. The cells were treated with vehicle or phendione for 3 h before collection.

To establish whether ATM activation plays a role in phendione-mediated growth suppression, we measured the ability of A375 and A375 BRAFi-resistant cell lines to form colonies after treatment with a specific ATM inhibitor (ATMi, KU60019). While treatment with ATMi alone did not significantly affect cell growth, ATMi significantly attenuated the growth inhibition incurred by phendione treatment in both cell lines tested (Fig. 6A,B; Supplemental Fig. S5A,B). Additionally, combined dose response of phendione and ATMi revealed antagonism between the two drugs, pointing to activation of ATM by phendione as a key component of the cytotoxic response (Fig. 6C,D). Consistent with this contention, inhibition of ATM or its depletion abrogated the phendione-mediated ATM-induced phosphorylation of CHK2 and γH2AX in both cell lines (Fig. 6E,F). Importantly, inhibition of ATM or its depletion did not affect phendione-mediated induction of MAPK response (Fig. 6E,F). In summary, these experiments show that phendione, through its inhibition of PP2A-C, exerts a robust induction of ATM activity, triggering DNA damage signaling and ultimately the apoptotic cascade.

### Phendione inhibits growth of human tumors with NRAS or BRAF mutations

We next examined the therapeutic potential of the above findings by assessing phendione effectiveness in patient-derived melanoma xenograft (PDX) models. Phendione was administered to NMRI nude mice via intraperitoneal (i.p) injection at 5 mg/kg body weight every other day alone or in combination with BRAFi every day for 20 d. Histopathological analysis of trachea, larynx, lungs, heart, esophagus, stomach, liver, gall bladder, pancreas, bone marrow, brain, and small intestine revealed no adverse reactions, suggesting lack of toxicity. Moreover, no significant weight loss was observed during the treatment period, indicating that phendione was well tolerated (data not shown).

**Figure 7.**
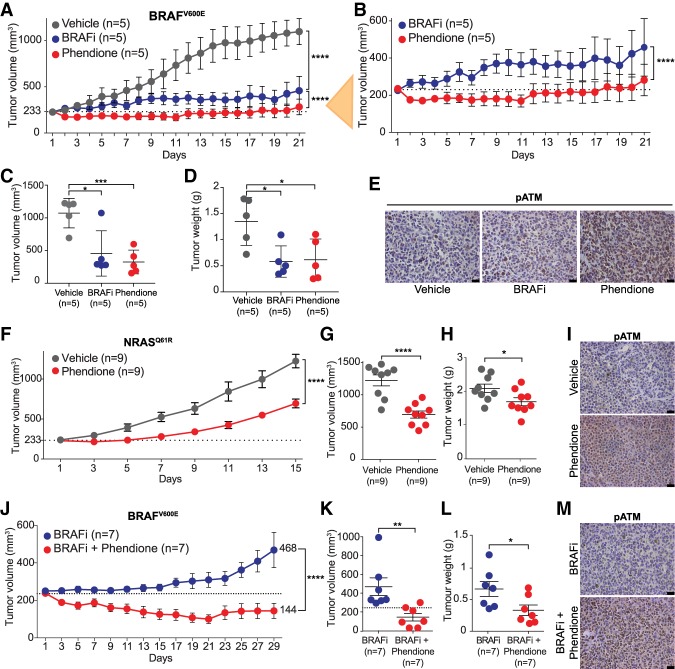
Phendione inhibits growth of human tumors with BRAF or NRAS mutations and overcomes resistance to BRAF inhibition in vivo. (*A*,*B*) Tumor volume of BRAF^V600E^ PDXs treated with BRAFi (dabrafenib) at 30 mg/kg daily by oral gavage, or phendione at 5 mg/kg every 2 d intraperitoneally (i.p.); vehicle was used as control in *A*. Zoomed-in curves of BRAFi and phendione treatments are shown in *B*. Data are mean ± SEM, *n* = 5. (****) *P* < 0.0001, by two-way ANOVA with Tukey's multiple comparisons test. Dotted line in *A* and *B* indicates the tumor volume on the day of treatment started. (*C*,*D*) Dot plots of BRAF^V600E^ PDX tumor volume (*C*) and weight (*D*) at day 21. Data are mean ± SEM. (*) *P* < 0.05; (***) *P* < 0.001; (****) *P* < 0.0001, by unpaired two-tailed Student's *t*-test. (*E*) Representative images of immunohistochemistry staining with antibody against phospho-ATM (S1981) for BRAF^V600E^ PDXs treated as indicated. (*F*) Tumor volume of NRAS^Q61R^ PDXs treated with phendione at 5 mg/kg every 2 d intraperitoneally (i.p.), or with vehicle. Data are mean ± SEM, *n* = 9. (****) *P* < 0.0001, by two-way ANOVA with Tukey's multiple comparisons test. (*G*,*H*) Dot plots of NRAS^Q61R^ tumor volume (*G*) and weight (*H*) at day 15. Data are mean ± SEM. (*) *P* < 0.05; (****) *P* < 0.0001, by unpaired two-tailed Student's *t*-test. (*I*) Representative images of immunohistochemistry staining with antibody against phospho-ATM (S1981) for NRAS^Q61R^ PDXs after indicated treatments. (*J*) Phendione prevents tumor relapse in BRAF^V600E^ PDX model. Data are mean ± SEM, *n* = 7. (****) *P* < 0.0001, by two-way ANOVA with Tukey's multiple comparisons test. (*K*,*L*) Dot plots of tumor volume (*K*) and weight (*L*) of BRAF^V600E^ PDXs. Data are mean ± SEM. (*) *P* < 0.05; (**) *P* < 0.01, by unpaired two-tailed Student's *t*-test. (*M*) Representative images of immunohistochemistry staining with antibody against phospho-ATM (S1981) of BRAF^V600E^ PDXs treated as indicated. Scale bars, 25 µm.

Next, we treated cohorts of melanoma PDXs harboring BRAF^V600E^ mutation with 5 mg/kg phendione every other day or 30 mg/kg dabrafenib daily, a BRAF^V600E^ inhibitor used in the clinic (Hauschild et al. 2012), and monitored tumor growth over time (Fig. 7A,B). Surprisingly, phendione treatment was significantly more effective in reducing tumor growth than the BRAF inhibitor (Fig. 7A–D). Importantly, phendione treatment resulted in the activation of ATM and induction of DNA damage pathway (Fig. 7E; Supplemental Fig. S6A). We next treated NRAS^Q61R^ driven melanoma PDXs with phendione, which resulted in a significant reduction in tumor volume and weight (Fig. 7F–H). Additionally, we observed a robust induction of ATM phosphorylation and the DDR (Fig. 7I; Supplemental Fig. S6B). These results attest to the effectiveness of phendione in reducing growth of melanomas with BRAF or NRAS mutations when used as a single agent in relevant preclinical models.

### Phendione overcomes resistance to BRAF inhibition in vivo

Finally, we evaluated whether combination treatment with a BRAF inhibitor and phendione could delay or prevent the development of resistance to BRAFi. We treated BRAF^V600E^ mutant melanomas in PDX cohorts with either BRAFi, or a combination of BRAFi and phendione (Fig. 7J). Notably, the combined treatment of BRAFi and phendione had a significantly greater inhibitory effect on tumor growth than the BRAFi alone, highlighting the potential of phendione to increase the efficacy of BRAFi. Additionally, this analysis revealed that while stable tumors begin to grow following 25 d of treatment with BRAFi alone, dual treatment with phendione sustained inhibition of tumor growth during the 29 d of the treatment (Fig. 7J–L). Similar to treatment with phendione alone, we observed a strong induction of ATM phosphorylation in tumors treated with phendione and BRAF inhibitor (Fig. 7M; Supplemental Fig. S6C). Finally, we treated parental A375 cells or BRAFi-resistant cells with combination of phendione and either BRAF or ERK1/2 inhibitors (Supplemental Fig. S6D,E). Interestingly, inhibition of ERK1/2 significantly antagonized the antiproliferative response of phendione in both parental and BRAFi-resistant A375 cells, consistent with the role of phendione in the inhibition of PP2A and subsequent ERK1/2 activation (Supplemental Fig. S6E). However, we observed a synergistic response following a combination treatment with BRAFi and phendione (Supplemental Fig. S6D), in agreement with the cooperative effect of phendione and BRAFi treatment in mouse models of melanoma (Fig. 7J–L). These results support the transient activation of ERK1/2 by phendione in the presence of BRAFi (Supplemental Fig. S6C) and point to ERK1/2 as a key target of PP2A in the negative regulation of MAPK pathway. Taken together, these experiments underscore the utility of phendione in diminishing the resistance that develops during prolonged BRAFi treatment.

## Discussion

The key findings of our study are as follows. First, we highlight the utility of a small molecule, phendione, as an inhibitor of PP2A, and support the importance of PP2A in the DNA damage response. Second, we demonstrate that PP2A depletion or inhibition by phendione induces ATM activation and the DNA damage response, leading to growth inhibition of melanoma that display intrinsic and acquired resistance to MAPK inhibitors. Third, we show that phendione, as a single agent, profoundly diminishes the growth of human melanoma tumors containing BRAF or NRAS mutations. Fourth, a combination treatment with phendione and BRAF inhibitor prevents the emergence of acquired resistance to BRAF inhibition in a PDX model of melanoma. Finally, through inhibition of PP2A, we usher in a cancer treatment strategy that subverts tumor dormancy through the activation of signaling pathways that promote cellular proliferation.

Our study highlights a unique strategy termed “targeted chemotherapy” in which the activation of DNA damage signaling through PP2A inhibition induces cell death in treatment-resistant cancer cells without physical DNA breaks. Remarkably, PP2A inhibition maintains proliferative capacity by hyperactivating the MAPK pathway and preventing escape from the apoptotic output of DNA damage signaling. Therefore, the key finding of our study is the discovery that while PP2A inhibition induces DNA damage signaling and promotes cancer cell death, unlike conventional chemotherapeutic agents, it does not result in cancer cell growth arrest. Indeed, in contrast to cisplatin or etoposide, in which their prolonged exposure promotes cellular dormancy, treatment of resistant melanoma cells with phendione activates proliferative pathways thereby preventing cell cycle exit and escape from cell death. Targeting PP2A with phendione uncovers the potential of a cancer therapy with a lower rate of tumor recurrence than current conventional chemotherapy due to the absence of cellular dormancy. Taken together, we propose phosphatase-directed therapy as an effective strategy to treat tumors that display resistance to targeted therapies and for those driven by constitutive RAS activation, for which there are no approved targeted therapies.

## Materials and methods

### Compounds

Vemurafenib (S1267), trametinib (S2673), PD0325901 (S1036), SCH772984 (S7101), etoposide (S1225), and cisplatin (S1166) were purchased from Selleck Chemicals. Phendione (1,10-phenanthroline-5,6-dione; #496383), N,N,N′,N′-tetrakis(2-pyridylmethyl)ethylenediamine (TPEN; #P4413), Deferoxamine mesylate (DFOM; #D9533), 3-hydroxy-1,2-dimethyl-4(1H)-pyridone (deferiprone [DFP] #379409), hydrogen peroxide solution (#95321), and pyrene (#82648) were purchased from Sigma. Ku-60019 (#17502) was purchased from Cayman Chemical. Dabrafenib was purchased from Biorbyt. LB-100 (#T2068) was purchased from TargetMol.

### Cell line and antibody

Primary human melanocytes HEMn were ordered from Thermo Fisher Scientific (#C-102-5C) and cultured according to the manufacturer's protocol. Primary patent derived melanoma lines MM029, MM034, MM047, MM057, and MM099 were from Dr. Jean-Christophe Marine laboratory. All other cell lines were ordered from ATCC and cultured with the suggested protocol. The antibodies against pATM-S1981 (#13050), pCHK2-T68 (#2661 and #2197), cleaved PARP (Asp214; #5625), pERK-T202/Y204 (#4370), ERK1/2 (#4696), pRSK-S380 (#9335), RSK1 (8408), cleaved caspase-3 (#9664), MYC (#18583), E2F1 (#3742), P21 (#2947), PP2A-B (the regulatory subunit of PP2A [PR55]; #4953), PP2A-A (#2041), PP2A-C (#2159), and PP5 (#2289) were ordered from Cell Signaling Technology. The antibodies against PP4-C (PPP4C; #PA5-96059) and PP6-C (PPP6C; #PA5-28919) were ordered from Thermo Fisher Scientific. The antibodies against CHK2 (#05-649), pATM-S1981 (#05-740), and γH2AX (Ser139; #06-636-I) were ordered from Sigma. The antibody against PARP1 (#39561) was ordered from Active Motif. The antibodies against PP1-Cα isoform (PPP1CA; #A300-904A), PP1-Cβ isoform (PPP1CB; #A300-905A), and PP1-Cγ isoform (PPP1CC, #A300-906A) were ordered from Bethyl. The antibodies against ATM (sc-377293) and GAPDH (sc-25778) were ordered from Santa Cruz Biotechnology.

### Immunofluorescence

Cells were seeded in 24-well plates on glass coverslips (Electron Microscopy Science) and treated as indicated. Before fixation with cold methanol for 20 min at −20°C, cells were washed twice with phosphate-buffered saline (PBS). Fixed cells were treated with cold acetone for 5 min at −20°C and then washed three times in PBS, and blocked with blocking solution (3% bovine serum albumin) for 30 min at room temperature. Fixed cells were incubated with primary antibodies as indicated in 1% bovine serum albumin overnight at 4°C, washed three times with PBS, and Alexa fluor 488- or Alexa fluor 568-labeled secondary antibody was added at a 1:1000 dilution for 1 h at room temperature). After three washes with PBS, the cells were mounted with ProLong Gold antifade mountant with DAPI (Thermo Fisher Scientific P36962). Samples were imaged with a Nikon A1plus Eclipse Ti2 microscope with a 60× plan apo 1.4 NA, at a 1-µm z depth interval, and a focused image was created using the z stack and Nikon Elements 5.02 software. Signals were quantified as number of foci per cell or integrated signal per cell. One-hundred-fifty cells from three independent experiments were analyzed.

### Cell cycle analysis

To determine the effect of the compounds on cell cycle progression, cells were treated with vehicle, phendione at 0.2 µM, or cisplatin at 2.5 µM for the indicated time. Cells were collected and fixed with cold 70% ethanol. After two washes with PBS, cells were suspended in staining buffer (PBS with 100 μg/mL RNase A, 50 μg/mL propidium iodide, 0.1% Triton X-100). Cells were stained for 2 h at room temperature. Flow cytometric analyses were performed using BD LSR Fortessa cell analyzer (BD Biosciences). Data were analyzed using FlowJo software.

### Protein and ligand preparation

We used the catalytic subunit of the protein phosphatase 2A holoenzyme as the target (PDB code 2iae chain C, region 4–308, protein length 309) (Cho and Xu 2007). We protonated the protein at pH 7, and added atom radii and Gasteiger partial charges per atom (net charge = −8, net charge including ions = −4) using PDB2PQR (v. 2.1.0) (Dolinsky et al. 2007) and the amber force field (Wang et al. 2000). The 3D structure of ligands were retrieved from ZINC15 (Sterling and Irwin 2015) (phendione code ZINC1580384, net charge = 0, HB acceptors = 4, and pyrene code ZINC1758808, net charge 0, HB acceptors = 0). We added Gasteiger charges to each ligand and refined the structures using the MMTK module (Hinsen 2000) in chimera (Pettersen et al. 2004) to obtain a planar conformation (number of steepest descent steps = 200, and conjugate gradient steps = 20). The catalytic site of PP2A was predicted to be druggable with Fpocket (Le Guilloux et al. 2009).

### Molecular docking

We used AutoDock tools (Morris et al. 2009) to prepare the docking files (protein and ligand). Next, we generated precalculated grid maps with AutoGrid (Morris et al. 2009) using a grid box of 126 × 126 × 126. We performed the flexible docking of phendione with Autodock 4.2 (Morris et al. 2009) using the Lamarckian genetic algorithm with local search (number of independent docking runs set to 1000 for reaching convergence). The rest of Autodock parameters were kept as default. Docking solutions were clustered with a tolerance of 2 Å and a representative solution per cluster was selected. We prepared the molecular graphics of the complex with the lowest estimated free energy of binding using Chimera (Pettersen et al. 2004). Protein-ligand interactions were analyzed with the protein–ligand interaction profiler (PLIP) using default parameters (Salentin et al. 2015).

### Phosphatase profiling

The effect of phendione on the activity of protein phosphatases was determined using a fluorescent-based in vitro enzymatic assay. The enzymatic reactions were conducted in duplicate at room temperature in a solution containing phosphatase enzyme and phendione at various concentrations in 25 mM HEPES (pH 7.5), 5 mM MgCl_2_, 0.01% Brij-35, 1 mM DTT, 1% DMSO, and 10 µM DiFMUP (6,8-difluoro-7-hydroxy-4-methylcoumarin). The indicated enzyme and substrate were prepared in fresh reaction buffer. Enzyme solution was delivered into the reaction well and then compounds in 100% DMSO were added to the enzyme solution using Echo 550 liquid handler (nanoliter range; Labcyte, Inc.). After 20 min of incubation at room temperature, the substrate solution was delivered into the reaction well to initiate the reaction. Phosphatase activity was monitored as a time-course measurement of the increase in fluorescence signal in the fluorescent substrate at an excitation of 355 nm and an emission of 460 nm using a microplate reader. The initial linear portion (0–40 min) of the slope (signal/min) was analyzed and the percentage of enzyme activity was calculated. Control in the absence of inhibitor was considered as 100% activity. Graphpad Prism was used to perform curve fitting and IC50 calculation. IC50 values were determined by the concentration causing a half-maximal percent activity. Pyrene was used as negative control.

### Cell viability and proliferation assay

To measure the effect of the compounds on cell proliferation, cells were plated (3000–5000 per well) in 96-well black plates with clear bottom and incubated overnight at 37°C in 5% CO_2_ before the drug treatment. Cell viability was measured 96 h after drug treatment using PrestoBlue (Invitrogen A13261). GraphPad Prism software was used to generate dose response curves and calculate IC50 values. Each experiment was repeated at least three times. To determine the effect of RNAi on cell proliferation, cells were trypsinized and the cell number was counted using Moxi Z mini automated cell counter (Orflo Technologies).

### Phendione derivative biotin labeling and pull-down

Phendione derivative (Alk-Phen) (structure shown in Supplemental Fig. S4) was custom-synthesized. Cells were treated with 5 µM Alk-Phen, 3 µM phendione, or DMSO for 3 h. Whole-cell lysate was obtained using lysis buffer containing 150 mM NaCl, 1.0% NP-40, and 50 mM Tris-HCl (pH 8.0) and dialyzed against buffer containing 10 mM NaCl, 1.0% NP-40, and 50 mM Tris-HCl (pH 8.0). The cell lysate was then clicked to biotin by adding 50 μM biotin picolyl azide (Click Chemistry Tools 1167), a preformed complex of CuSO_4_:THPTA (1.0 mM:5.0 mM), and 5 mM sodium ascorbate, sequentially. The click reaction was performed for 30 min at room temperature protected from light. The cell lysate was then dialyzed against lysis buffer to remove unclicked azidobiotin and pull-down was carried out with Dynabeads MyOne streptavidin C1 (Thermo Fisher Scientific 65002).

### Comet assay

A375 and A375 BRAFi-resistant cells were treated with 0.5 μM phendione for 8 h before harvesting. Vehicle (DMSO) and etoposide at 20 μM were included as negative and positive controls, respectively. OxiSelect comet assay kit (Cell Biolabs, Inc., STA-352) was used to assess DNA damage in the cells. In brief, cell suspension at 1 × 10^5^/mL was prepared in ice-cold PBS (without Mg^2+^ and Ca^2+^). Cell samples were prepared by mixing cell suspension with comet agarose (37°C) at 1:10 ration (v/v) and 75 µL was immediately transferred onto the top of comet agarose base layer. Cells were treated with prechilled lysis buffer for 45 min at 4°C and then treated by alkaline solution for 30 min. The comet agarose was subjected to alkaline electrophoresis for 15–30 min at 1 V/cm with 300 mA current and then washed three times with distilled water. The comet agarose was stained with diluted Vista Green DNA dye for 15 min at room temperature after being fixed with cold 70% ethanol for 5 min. Images were taken with an epifluorescent microscope using a FITC filter. Olive tail moment was calculated using automated ImageJ based plugin OpenComet (v1.3, available at http://www.cometbio.org). Data represent 150 cells analyzed from three independent experiments.

### Drug combination effect

To test the effect of drug combination, cells were plated (3000–5000 per well) in 96-well black plates with clear bottom and incubated overnight at 37°C in 5% CO_2_ before the drug treatment. Cells were treated with various concentrations of phendione in combination with various concentrations of ATM, BRAF, or ERK inhibitor. Cell viability was measured 96 h after drug treatment using PrestoBlue (Invitrogen A13261). The SynergyFinder Web application (https://synergyfinder.fimm.fi) was used to calculate synergy scores and generate 2D synergy maps.

### siRNA transfection

siRNA transfection was performed using RNAiMAX transfection reagent (#13778150) and Silencer Select predesigned siRNA from Thermo Fisher Scientific following manufacturer's instructions. Negative control no. 1 siRNA (AM4635), siRNAs against PP2A-Cα isoform (PPP2CA; ID s10959), PP2A-Cβ isoform (PPP2CB; ID s10962), PP2A-Aα isoform (PPP2R1A; ID s10964), PP2A-Aβ isoform (PPP2R1B; ID s10968), PP1-Cα isoform (PPP1CA; ID s10931), PP1-Cβ isoform (PPP1CB; ID s10934), PP1-Cγ isoform (PPP1CC; ID s720), PP4-C (PPP4C; ID s10999), PP6-C (PPP6C; ID s11016), and ATM (ID s530445) were used.

### Colony formation assay

Log-phase cells were treated with vehicle, or with increasing concentrations of phendione in the absence or presence of 5 µM ATM inhibitor for 3 h. After treatment, a certain number of viable cells were plated into 100-mm culture dishes to yield 50–200 surviving colonies. Colonies were grown for 10–14 d, after which they were fixed with methanol and stained with 1% crystal violet. The number of colonies was normalized to the number of cells plated to calculate the surviving fraction. Each experiment was performed three times.

### Detection of mitotically quiescent cells in vitro

Cells were labeled with Vybrant DiD cell-labeling solution (Thermo Fisher Scientific V-22887) for 20 min according to manufacturer's instructions and then treated with 0.2 µM phendione, 2.5 µM cisplatin, and 5 µM etoposide for 3 h. DMSO (vehicle) was used as control. Cells were maintained in growing medium after drug treatment and kept in incubator following routine cell culture protocols. On day 14 after drug treatment, the cells were pulse-chased with bromodeoxyuridine (BrdU) and immunofluorescent staining of incorporated BrdU was performed using FITC BrdU flow kit (BD BD Biosciences 559619) following manufacturer's protocol. Retention of Vybrant DiD cell-labeling dye and BrdU incorporation were measured using CytoFLEX LX flow cytometer (Beckman Coulter Life Sciences). Data were analyzed with FlowJo software.

### Annexin V apoptosis flow cytometry

After trypsinized and washed with PBS, the cells were stained with Annexin V apoptosis detection kit according to the manufacturer's protocol (BD BD Biosciences 556547 and 559763), and then analyzed with CytoFLEX LX flow cytometer (Beckman Coulter Life Sciences). Data were processed with FlowJo software.

### Intracellular zinc measurement

At the end of drug treatment, the cells were trypsinized and resuspended in Hank's balanced salt solution (HBSS). A final concentration of 20 µM zinquin ethyl ester (Cayman Chemical 15133) was added into cell suspension to stain Zn^2+^ in live cells. The cell suspension was incubated for 30 min at 37°C before flow cytometry analysis. Unstained cells were used as negative control.

### ROS production measurement

Total reactive oxygen species (ROS) production was measured with ROS assay kit (Thermo Fisher Scientific 88-5930-74). In brief, the cell suspension was pretreated with ROS stain for 30 min, followed by 3 h of drug treatment. Fluorescent intensity was measured by flow cytometry using FITC channel. Hydroxyl peroxide (H_2_O_2_; 200 µM) was used as positive control.

### In vivo tumor models (PDX)

Animals were housed under pathogen-free conditions. All procedures involving animals (NMRI nude, female, 4 wk old) were performed in accordance with the guidelines of the Catholic University of Leuven (KU Leuven) Animal Care and Use Ethical Committee (P147/2012). Mel018 and Mel006 PDX models were derived from two different metastatic melanoma lesions, carrying the NRAS^Q61R^ and BRAF^V600E^ mutations, respectively. Written informed consent was obtained from both patients and all procedures involving human samples were approved by the UZ Leuven/KU Leuven Medical Ethical Committee (ML8713/S54185).

In order to test the efficacy of phendione on tumor growth inhibition, mice were implanted subcutaneously with patient-derived melanoma tumors harboring NRAS^Q61R^ (Mel018) or BRAF^V600E^ (Mel006) mutation. Once tumors reached 200–300 mm^3^, mice were randomly divided into groups. For treatment with phendione only, Mel018 and Mel006 mice were treated with vehicle (DMSO) or phendione at 5 mg/kg every 2 d intraperitoneally (i.p.). For the combined treatment with the BRAF inhibitor, cohorts of Mel006 were treated with the BRAF inhibitor dabrafenib at 30 mg/kg, or vehicle administered daily by oral gavage, in addition to either vehicle or phendione at a concentration of 5 mg/kg, administered i.p. every 2 d. Tumor size was measured every 2 d and tumor volume was calculated using the formula l × w × h·π/6 (length × width × height × π/6). No specific randomization method was used. According to animal welfare guidelines, mice have to be sacrificed when tumors reach a volume of 2 × 10^3^ mm^3^ or when their body weight decreases >20% from the initial weight. Mice used in this work did not reach or overcome these limits. The investigators were blinded for the evaluation of the results.

At end point of the experiment, tumor tissue was collected for paraffin embedding, and then 5-µm tissue sections were prepared. Immunohistochemistry staining was performed with antibodies against phospho-ATM (S1981), phospho-CHK2 (T68), and γH2AX using IHC kit (BioVision, Inc. K405) according to manufacturer's instructions.

### Data availability

All data are available here or in the Supplemental Material. Additional data that support the findings of this study are available on request.

## Supplementary Material

Supplemental Material
