# Supplementary material for: Targeted chemotherapy overcomes drug resistance in melanoma
Source: Genes Dev. 2020 May 1;34(9-10):637–49. doi: 10.1101/gad.333864.119 (PMC7197350; doi:10.1101/gad.333864.119)
Supplement: Supplemental Material [file supp_34_9-10_637__index.html]

Targeted chemotherapy overcomes drug resistance in melanoma — Supplemental Material 

# Targeted chemotherapy overcomes drug resistance in melanoma

## Supplemental Material

- Supplemental\_Figure\_Legends.docx
- Supplemental\_Fig\_1.pdf
- Supplemental\_Fig\_2.pdf
- Supplemental\_Fig\_3.pdf
- Supplemental\_Fig\_4.pdf
- Supplemental\_Fig\_5.pdf
- Supplemental\_Fig\_6.pdf
