# Supplementary material for: Targeted chemotherapy overcomes drug resistance in melanoma
Source: Genes Dev. 2020 May 1;34(9-10):637–49. doi: 10.1101/gad.333864.119 (PMC7197350; doi:10.1101/gad.333864.119)
Supplement: Supplemental Material [file supp_gad.333864.119_Supplemental_Figure_Legends.docx]

**Supplemental Figures**

**Supplemental_Fig_S1. Downregulation of PP2A and phendione treatment inhibits cell proliferation and induces apoptosis. *(A-D)*** Growth inhibition curves and IC50 values of melanoma cell line A375 (BRAFV600E) and its derived cell lines with adaptive resistance to BRAF inhibitor (Vemurafenib) (A375 BRAFi resistant) or to both BRAF and MEK inhibitors (Vemurafenib and Trametinib) (A375 BRAFi+MEKi resistant) were treated with *(****A****)* BRAF inhibitor, *(****B****)* MEK inhibitor, *(****C****)* ERK inhibitor, or *(****D****)* phendione for 96 hours. Data are presented as mean ± SD of three independent experiments. ***(E)*** Apoptosis measured by Annexin V staining flow cytometry in parental A375 and A375 BRAFi resistant cells 3 days after transfected with control siRNA or siRNAs targeting PP2A-C (siRNA pool against α and β isoforms) and PP2A-A (siRNA pool against α and β isoforms). ***(F)*** Cell cycle analysis performed with flow cytometry in A375 and A375 BRAFi resistant cells 3 days after transfected with siRNA as described in *(E)*.

**Supplemental_Fig_S2. Phendione inhibits PP2A and activates DNA damage signaling in melanoma cells.** *(****A****)* Overview of the binding model for phendione and pyrene. Docking pose of phendione (green) interacting with the catalytic ions (purple) at the active site of PP2A Cα, and pyrene (black) binding to an adjacent pocket within approx. 7 Å (dotted-lines represent the pairwise measurements). *(****B****)* Diagram showing phendione derivative structure and copper-catalyzed Click reaction. *(****C****)* Quantification of pATM, γH2AX, and pCHK2 signal after immunofluorescent staining. A375 BRAFi resistant cells were treated with vehicle, pyrene (0.5 μM), or phendione (0.5 μM) for 3 hours and then immunofluorescent staining was performed. Data are represented as mean ± SD of three independent experiments; 150 cells were analyzed in each condition. *(****D****)* Representative images of immunostaining of phospho-ATM (S1981, red), γH2AX (red) and phospho-CHK2 (Thr68, green). DAPI staining shows the location of the nucleus. Scale bars, 10 μm. ***(E, F)*** Apoptosis assay by flow cytometry. A375 *(****E****)* and A375 BRAFi resistant cells*(****F****)* were treated with vehicle or phendione for 24 hours, and then stained with Annexin V staining kit for flow cytometry.

**Supplemental_Fig_S3. Phendione induces mild G2/M accumulation and inhibits melanoma cell proliferation at submicromolar concentration.** *(****A****)* Histogram of cell cycle analysis of A375 and A375 BRAFi resistant cells after vehicle, cisplatin or phendione treatment as indicated. *(****B****)* Quantification of cell cycle analysis for A375 cells. Data shown are mean ± SD of three independent experiments. *p<0.05, **p<0.01. Unpaired two-tailed Student’s t-test was used for statistical analysis. ***(C)*** Histogram of reactive oxygen species (ROS) production measured by flow cytometry. ***(D)*** IC50 values measured after 4 days of treatment with vehicle and increasing concentration of phendione in the cell line panel. ***(E)*** Immunoblots of primary melanocyte cell lysate collected after 3 hours of treatment with drugs as indicated.

**Supplemental_Fig_S4. Phendione does not chelate ations in the cells. *(A, B)*** Immunoblots of whole cell lysate collected from A375 *(A)* and A375 BRAFi resistant cells *(B)* after treated for 3 hours with vehicle or phendione prepared in regular medium or medium containing containing 10 µM CuSO_4_ or 20 µM FeCl_3_. ***(C, D)*** Growth inhibition curves of A375 *(C)* and A375 BRAFi resistant cells *(D)* with treatment of phendione in regular medium or medium containing 20 mM of MgCl_2_, CaCl_2_ or NaCl for 96 hours. Data presented as mean ± SD of three independent experiments.

**Supplemental_Fig_S5. Effect of ATMi on colony formation.** Colony formation efficiency of *(****A****)* A375 and *(****B****)* A375 BRAFi resistant cells after treatment with ATM inhibitor KU-60019 (5 μM) or vehicle for 3 hours. Data are shown as mean ± SD of three independent experiments. ns, not significant by unpaired two-tailed Student’s t test.

**Supplemental_Fig_S6. Phendione treatment induces DNA damage signaling in tumor xenografts and combination treatment with BRAFi does not affect DNA damage signaling activation in cells, however, ERKi treatment antagonizes the drug efficacy of phendione. *(A, B)*** Representative images of immunohistochemistry staining with antibody against phospho-CHK2 (Thr68) and γH2AX of *(****A****)* BRAF^V600E^ and *(****B****)* NRAS^Q61R^ PDX xenografts after indicated treatments. Scale bars, 25 μm. ***(C)*** Immunoblots of A375 and A375 BRAFi resistant cell lysates after treatment with phendione, pyrene (3 μM), or vehicle for 3 hours in presence or absence of BRAF inhibitor (Vemurafenib, 1 μM). ***(D, E)*** Synergy maps for A375 and A375 BRAFi resistant cells showing the effect of combined treatment of phendione with BRAFi *(D)* or with ERKi *(E)* at indicated concentrations for 96 hours. The 2D synergy matrix was generated with SynergyFinder software using HAS model.
