# Supplementary figures and images for: Targeted chemotherapy overcomes drug resistance in melanoma
Source: Genes Dev. 2020 May 1;34(9-10):637–49. doi: 10.1101/gad.333864.119 (PMC7197350; doi:10.1101/gad.333864.119)

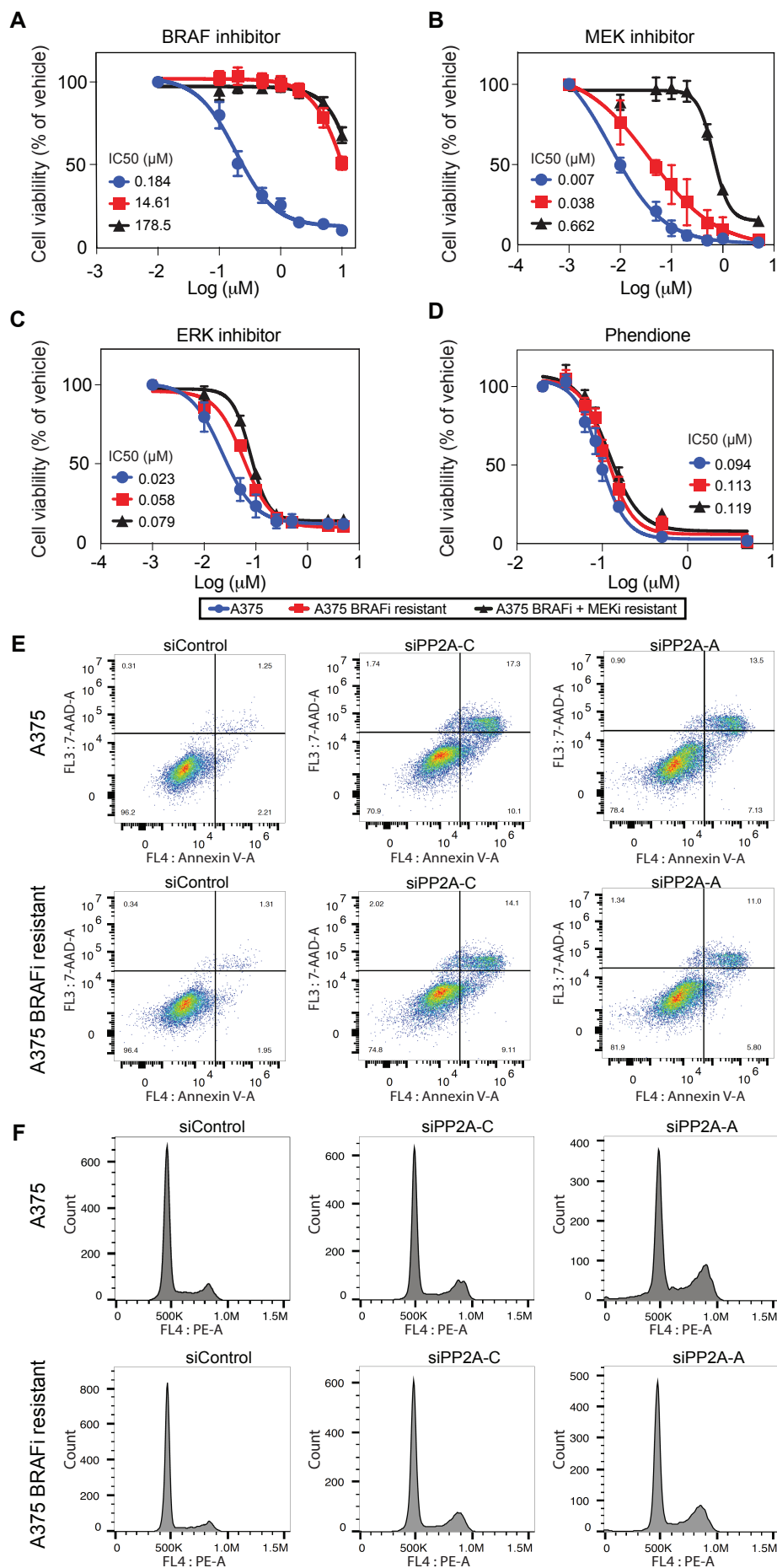

Supplement: Supplemental Material [file supp_gad.333864.119_Supplemental_Fig_1.pdf]

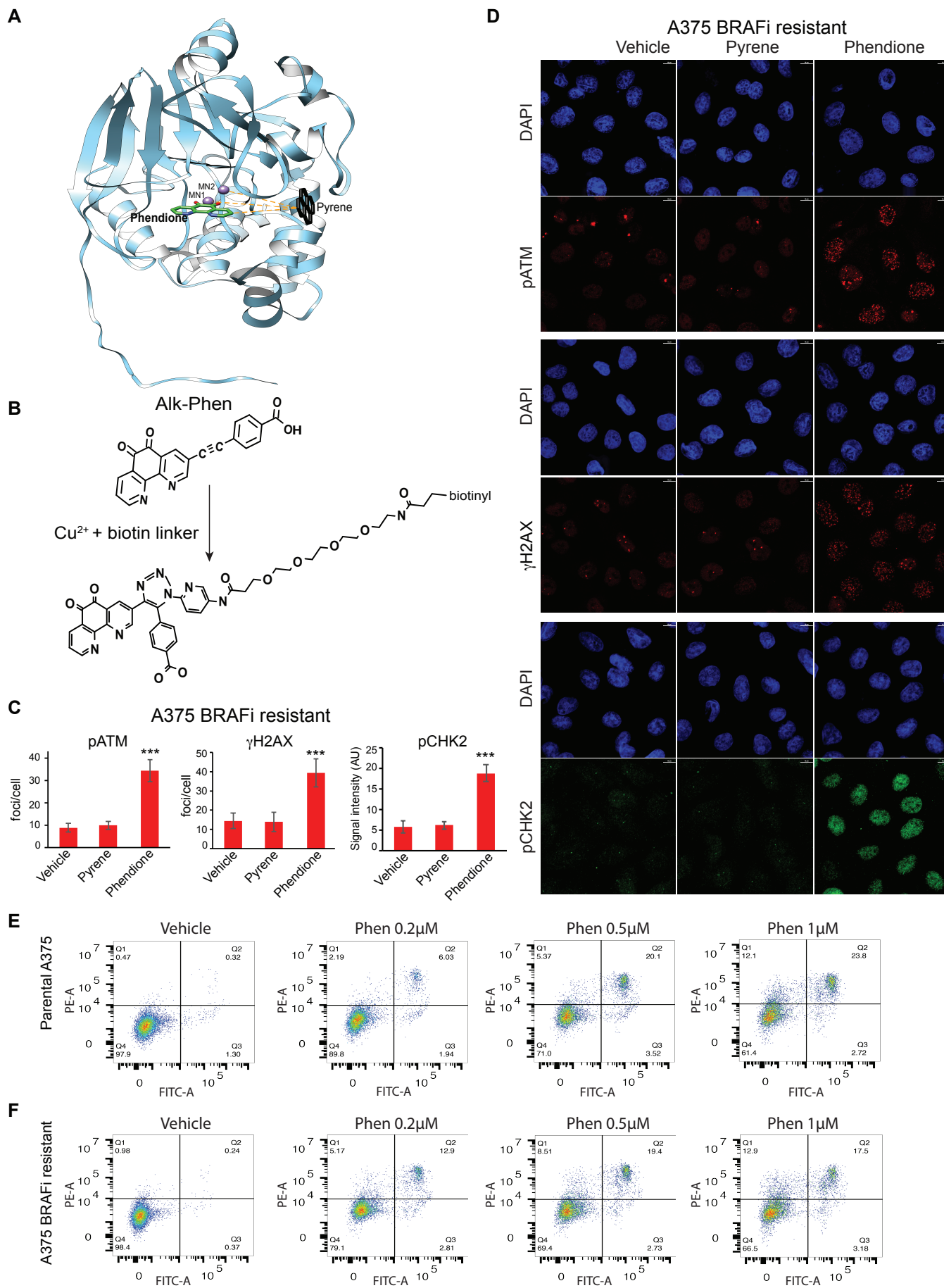

Supplement: Supplemental Material [file supp_gad.333864.119_Supplemental_Fig_2.pdf]

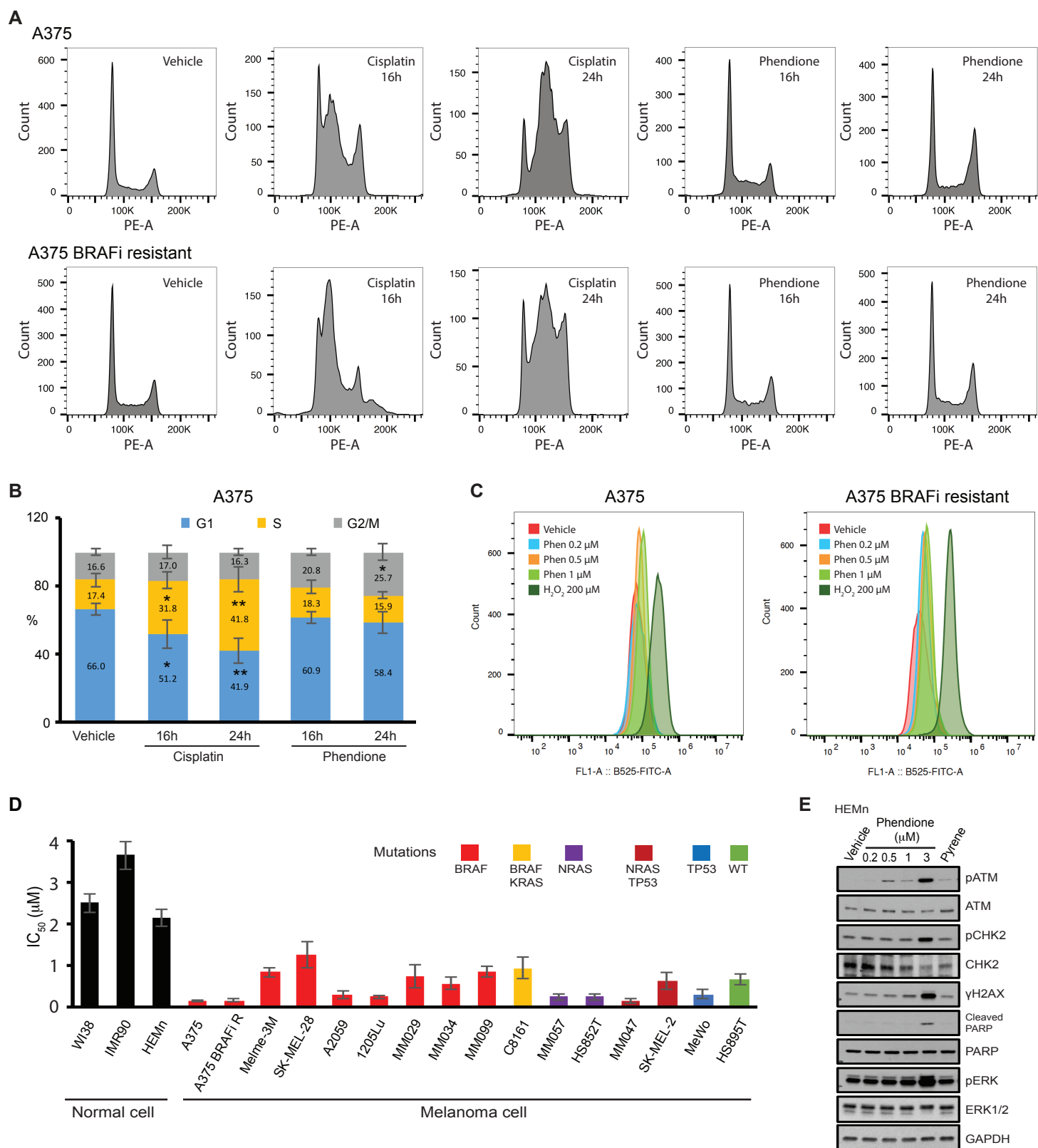

Supplement: Supplemental Material [file supp_gad.333864.119_Supplemental_Fig_3.pdf]

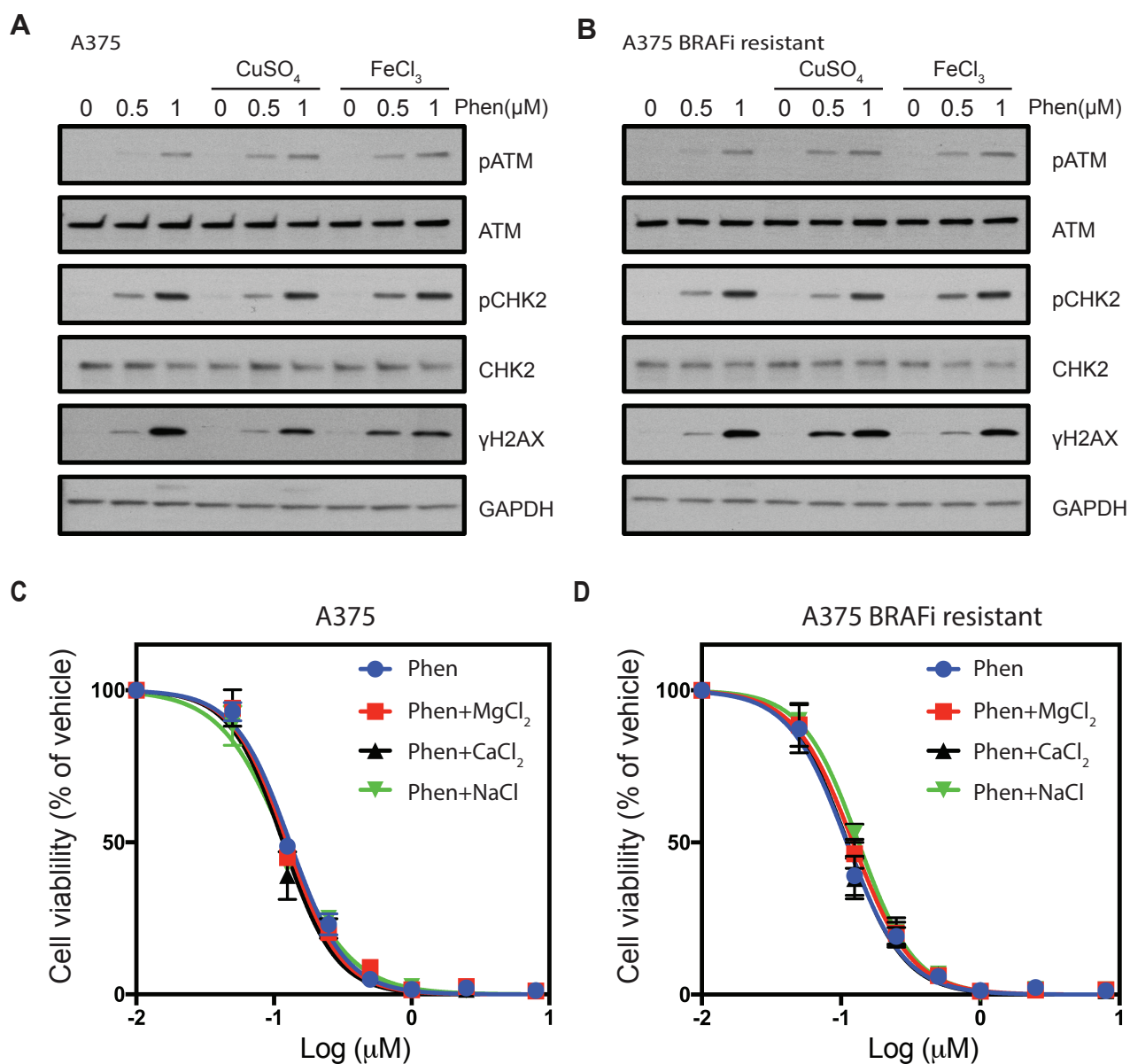

Supplement: Supplemental Material [file supp_gad.333864.119_Supplemental_Fig_4.pdf]

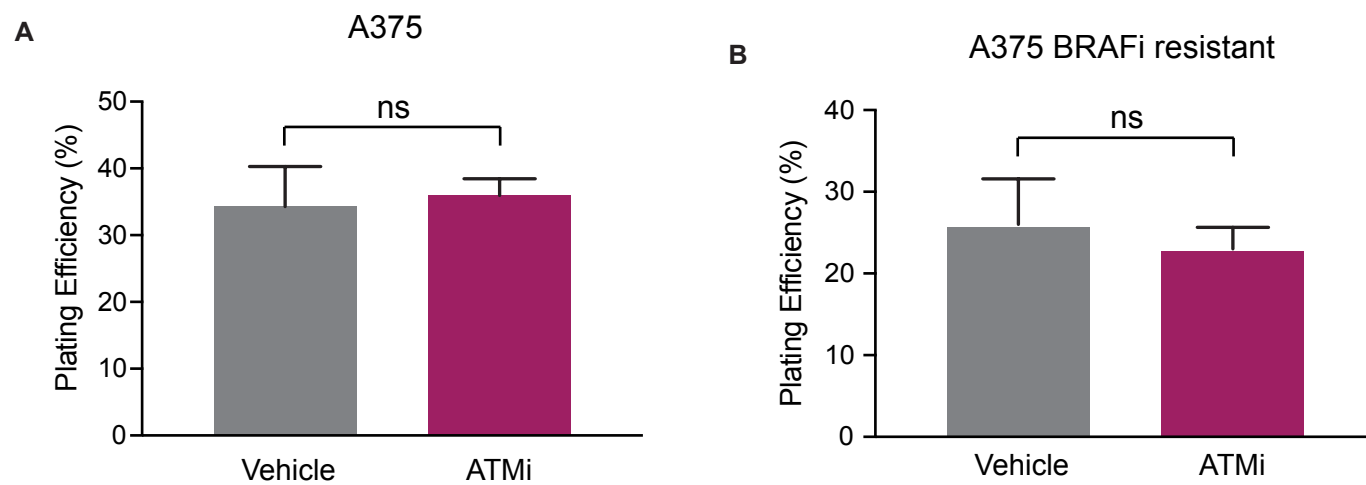

Supplement: Supplemental Material [file supp_gad.333864.119_Supplemental_Fig_5.pdf]

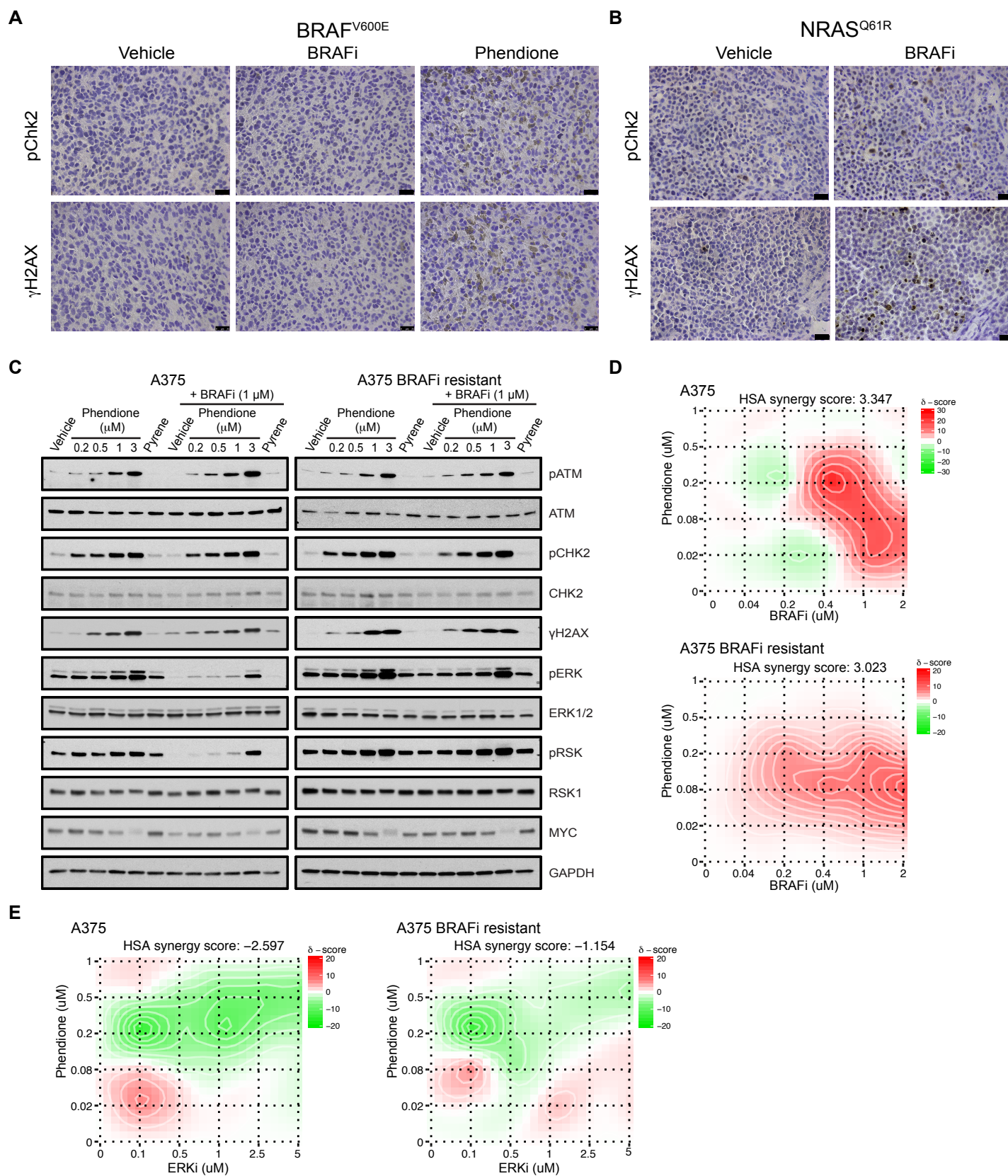

Supplement: Supplemental Material [file supp_gad.333864.119_Supplemental_Fig_6.pdf]
